# Predictive value of pan-immune-inflammation value in the prognosis of adults with status epilepticus: a retrospective study

**DOI:** 10.3389/fnagi.2025.1601816

**Published:** 2025-07-16

**Authors:** Jie Fu, Yifei Chu, Chenxin Zhao, Lilei Peng

**Affiliations:** ^1^Department of Neurology, The Affiliated Hospital of Southwest Medical University, Luzhou, China; ^2^Department of Neurosurgery, The Affiliated Hospital of Southwest Medical University, Luzhou, China; ^3^Clinical Medical College, Southwest Medical University, Luzhou, China

**Keywords:** pan-immune-inflammation value, status epilepticus, inflammation, biomarker, prognosis

## Abstract

**Objective:**

To investigate the predictive capacity of the pan-immune-inflammation value (PIV) for functional outcomes in patients with status epilepticus (SE).

**Methods:**

In this study, we investigated and confirmed the prognostic significance of PIV in adult patients with SE. Clinical information and laboratory test data of the patients were extracted. We gathered the information on 30-day mortality following SE and used the modified Rankin scale (mRS) to assess functional prognosis. Multivariable logistic regression models were employed to assess the relationship between PIV and SE prognosis. Additionally, receiver operating characteristic (ROC) curve analysis was conducted to identify the optimal PIV threshold for predicting poor outcomes of SE patients.

**Results:**

Initially, the discovery cohort comprising 132 SE patients were examined. The findings revealed that 18.2% (24/132) of patients died within a 30-day period post-SE, and 25.8% (23/89) experienced unfavorable prognosis during the 6-month follow-up period. Multivariate logistic regression analysis showed that higher PIV on admission was independently related to poor 6-month prognosis of SE patients (odds ratio: 1.002; 95% confidence interval, 1.000–1.004; *p* = 0.026). ROC analysis determined 1,090 as the optimal cutoff value of PIV for predicting poor 6-month prognosis in these patients. Moreover, multivariate logistic regression analysis of the external cohort demonstrated that PIV ≥ 1,090 was an independent predictor for poor SE outcome (odds ratio: 42.433; 95% confidence interval, 1.456–1236.343; *p* = 0.029), which verified our findings.

**Conclusion:**

Higher PIV is strongly correlated with an elevated risk of unfavorable SE prognosis. Our results suggest that PIV is a simple and reliable predictor for SE prognosis.

## Introduction

Status epilepticus (SE) is a critical neurological emergency, which is defined as a disorder caused by either the failure of seizure termination or the activation of related mechanisms that induce long-lasting seizures (Kapur, 2025). SE can destroy brain function, and lead to permanent neurological impairment (Specchio and Auvin, 2025). SE has been reported to occur in 10 ~ 41 per 100,000 people every year and affect people of any age (Lin et al., 2024). SE is often involved in a variety of complications including brain edema, aspiration pneumonia, electrolyte disturbance, and cardiac arrhythmia, which places a great clinical and economic burden (Shi et al., 2024; Farjoud Kouhanjani et al., 2025). Therefore, developing efficacy indicators for predicting SE prognosis is essential to stratify SE patients. Critical SE patients at high risks of adverse outcomes could benefit from early escalation of antiepileptic therapies, constant video-electroencephalogram monitoring and neuroprotective treatments, while overtreatment could be carefully avoided for patients with favorable outcomes, which contributes to optimizing medical care and reducing costs.

Accumulating evidence indicates that inflammation and SE affect each other. SE could activate astrocytes and microglia through inducing receptors on their cell surfaces. Furthermore, activated glial cells could not only produce massive inflammatory mediators but also upregulate N-methyl-D-aspartate (NMDA) receptors of postsynaptic cells to facilitate seizure recurrence (Foiadelli et al., 2023). Several studies have demonstrated that various inflammation markers could be used to predict SE prognosis, such as albumin (Misirocchi et al., 2025) and procalcitonin (PCT) (Sutter et al., 2015). Among the inflammation markers, those derived from routine blood tests may hold broad clinical application prospects, due to their simplicity, rapidness and cost-effectiveness. The pan-immune-inflammation value (PIV), as a new inflammation parameter integrating neutrophil, monocyte, platelet, and lymphocyte counts, has emerged as a potential indicator of systemic inflammation and immune status (Wang et al., 2025). PIV is reported to be correlated with clinical outcomes of multiple disorders, including cardiovascular diseases (Murat et al., 2024), cerebrovascular diseases (Wang D. et al., 2023; Wang S. et al., 2023), and rheumatoid arthritis (Mardan et al., 2025). Notably, recent studies have found that compared to other inflammatory parameters including systemic immune inflammation index (SII), neutrophil-to-lymphocyte ratio (NLR), and platelet-to-lymphocyte ratio (PLR), PIV is a better predictor (Fucà et al., 2020; Murat et al., 2023). Interestingly, Shi et al. (2024) observed a temporary inflammatory reaction occurring at the acute stage of adult patients with convulsive SE, as displayed by multiple inflammatory markers including PIV increasing during acute seizures and returning to baseline levels after remission. Besides, a study from Şahin and Güneş (2025) found that PIV might be related to the development of SE to refractory SE. Despite that these data provided a possible relationship between PIV and SE, it has not yet been identified whether PIV can predict functional outcome post-SE onset. Hence, the present research sought to examine the association of admission PIV and 30-day mortality and poor 6-month prognosis after SE.

## Methods

### Study population and patient selection

We retrospectively reviewed patients with SE admitted to the affiliated hospital of Southwest Medical University from January 2020 to July 2024 and to the affiliated Traditional Chinese Medicine hospital of Southwest Medical University between January 2022 and July 2024. The former cohort represented the discovery cohort, while the latter served as an external validation cohort. The present research included individuals aged more than 18 years old who were diagnosed with SE. SE referred to generalized tonic–clonic seizures lasting more than 5 min or without recovery of consciousness between seizures, focal seizures with impaired consciousness lasting over 10 min, and absence seizures persisting beyond 10–15 min (Trinka et al., 2015). We excluded patients whose SE was attributed to hypoxic–ischemic encephalopathy following cardiac arrest, as well as those with incomplete clinical data. The research received approval from the ethics committee of Southwest Medical University (Approval No. KY2024478), and it was performed in compliance with the Helsinki Declaration principles. All patients or their family representatives gave informed consent.

### Data collection

Demographics and clinical characteristics included sex, age, SE etiology, history of previous seizures, status epilepticus severity score (STESS) at SE onset, admission modified Rankin scale (mRS) scores, Charlson comorbidity index, and the number of antiepileptic drugs (AEDs). Etiology of SE was classified into four groups as proposed by the International League Against Epilepsy (ILAE): acute symptomatic SE, remote symptomatic SE, progressive symptomatic SE, or SE of unknown etiology (Sutter et al., 2015). STESS scale includes four components: age, prior seizures, type of seizure and consciousness status, which is applied to grade SE severity (Rossetti et al., 2006). Data of laboratory tests were collected within the first 24 h post-admission, including hemoglobin, neutrophil count, monocyte count, lymphocyte count, platelet count, and albumin. The blood-based neutrophil-to-albumin ratio (NAR), neutrophil-to-lymphocyte ratio (NLR), platelet-to-lymphocyte ratio (PLR), and monocyte-to-lymphocyte ratio (MLR) were defined as the ratio of neutrophil count to albumin level, the ratio of neutrophil count to lymphocyte count, the ratio of platelet count to lymphocyte count and the ratio of monocyte count to lymphocyte count, respectively. The PIV was calculated according to the following formula: neutrophil count × monocyte count × platelet count/lymphocyte count (Shi et al., 2024). Follow-up data were obtained through telephone interviewing or reviewing the medical records of patients. The information of death within 30 days post-SE onset was recorded, and subsequently surviving patients received follow-up until 6 months post-SE onset. Patients’ clinical outcomes at 6 months post-SE were evaluated using mRS scores. Poor prognosis was defined as an mRS score ≥ 3 (including death), while good prognosis was regarded as an mRS score of less than 3 (Qiao et al., 2022).

### Statistical analysis

Categorical variables were described as counts (percentage) and analyzed with the chi-square (χ2) test. Continuous variables of normal distribution were presented as means (standard deviation) and analyzed with the t-test, while continuous variables of skew distribution were expressed as medians (interquartile range, IQR) and compared with the Mann–Whitney U-test. Variables with *p* < 0.10 in the univariate analysis were selected as the primary covariates and entered into the multivariate logistic regression model. We applied the variance inflation factors (VIF) to assess multicollinearity. The variables with tolerance > 0.1 and VIF < 5 were selected for further multivariate analysis. Receiver operating characteristic (ROC) curve analysis was employed to analyze the predictive ability of markers for clinical outcomes in SE patients, and the DeLong test was utilized to analyze the areas under ROC curves. The Youden index was used to determine the cutoff point of PIV. We applied GraphPad Prism 9.0 software, SPSS 26.0 software and MedCalc 22.0 software to analyze all data. Statistical significance was defined as *p* < 0.05.

## Results

### Baseline characteristics

25 patients who suffered from hypoxic–ischemic encephalopathy, 13 with incomplete clinical information as well as 17 who failed to follow up were excluded in the Discovery cohort. Finally, 132 patients with SE were included. Out of the 132 patients with SE, 24 died and 108 survived within 30 days post-SE. Among these 108 surviving patients, 6-month post-SE follow-up data were available for 89 patients (82.4%). Based on the mRS score at 6 months post-SE onset, 25.8% (23/89) had an unfavorable prognosis, and 74.2% (66/89) of patients had a favorable prognosis. Moreover, 55 SE patients meeting our eligibility criteria and finishing follow-up assessments were included in the external validation cohort. Among the 55 patients, 39 (70.9%) possessed good outcomes, and 16 (29.1%) suffered from adverse outcomes.

### PIV and 30-day mortality post-SE

Table 1 demonstrated the univariable analyses of clinical characteristics and laboratory data between survivors and non-survivors. Compared to survivors, non-survivors possessed remarkably higher baseline mRS scores, STESS, Charlson comorbidity index, neutrophil count, NLR, MLR, NAR and PIV (all *p* < 0.05). In addition, we found lower levels of lymphocyte count and albumin in non-survivors compared to survivor patients (Both *p* < 0.05). No obvious difference was observed in sex, age, etiology of SE, ratios of SE induced by infection or stroke, the number of AEDs, prior seizures, hemoglobin, monocyte count, platelet count and PLR between non-survivors and survivors (all *p* > 0.05). Furthermore, variables with a *p*-value of less than 0.10 in the univariate analysis were incorporated into the multivariate logistic regression model. The multivariate analysis indicated no obvious association of PIV with 30-day death of SE patients [odds ratio: 1.000; 95% confidence interval, 0.999–1.001; *p* = 0.768] (Table 2).

**Table 1:** Univariate analysis of clinical characteristics and laboratory data between survivors and non-survivors.

Variable	Survivors (*n* = 108)	Non-survivors (*n* = 24)	*p*
Male (*n*, %)	66.0 (61.1)	12.0 (50.0)	0.317
Age, years, median (IQR)	51.0 (28.8–64.5)	64.0 (40.8–71.3)	0.120
SE etiology grouped according to the ILAE (*n*, %)			0.093
Acute symptomatic seizures	34.0 (31.5)	14.0 (58.3)	
Remote symptomatic unprovoked seizures	20.0 (18.5)	2.0 (8.3)	
Symptomatic seizures due to progressive CNS disorders	10.0 (9.3)	1.0 (4.2)	
Seizures of unknown etiology	44.0 (40.7)	7.0 (29.2)	
Stroke etiology of SE (*n*, %)	9.0 (8.3)	5.0 (20.8)	0.152
Infectious etiology of SE (*n*, %)	16.0 (14.8)	7.0 (29.2)	0.168
No history of seizures (*n*, %)	65.0 (60.2)	18.0 (75.0)	0.174
mRS baseline, median (IQR)	1.5 (1.0–4.0)	4.0 (4.0–5.0)	< 0.001
STESS at SE onset, median (IQR)	2.0 (2.0–3.0)	4.0 (3.0–5.3)	< 0.001
Charlson comorbidity index, median (IQR)	1.0 (0–2.0)	3.0 (2.0–4.0)	< 0.001
AEDs, median (IQR)	3.0 (2.0–3.0)	3.0 (2.8–4.0)	0.133
Hemoglobin (g/L), mean (SD)	135.2 (20.7)	134.0 (25.7)	0.813
Neutrophil count (x10^3^ /μL), median (IQR)	6.7 (4.1–9.3)	9.4 (6.5–11.8)	0.003
Lymphocyte count (x10^3^ /μL), median (IQR)	1.3 (1.0–2.0)	1.0 (0.6–1.4)	0.031
Monocyte count (x10^3^ /μL), median (IQR)	0.5 (0.4–0.7)	0.6 (0.4–0.7)	0.149
Platelet count (x10^3^ /μL), median (IQR)	207.0 (169.0–266.0)	192.0 (132.8–256.5)	0.261
Albumin (g/L), mean (SD)	42.3 (5.5)	38.1 (7.5)	0.002
Neutrophil to lymphocyte ratio, median (IQR)	3.9 (2.2–7.9)	8.5 (4.7–13.9)	0.003
Monocyte to lymphocyte ratio, median (IQR)	0.3 (0.2–0.5)	0.5 (0.3–1.0)	0.016
Platelet to lymphocyte ratio, median (IQR)	150.8 (94.4–218.9)	174.2 (113.2–288.0)	0.281
Neutrophil to albumin ratio, median (IQR)	0.1 (0.1–0.2)	0.2 (0.2–0.3)	0.001
PIV, median (IQR)	444.4 (189.2–973.9)	599.2 (442.9–2286.1)	0.007

IQR, interquartile range; ILAE, International League Against Epilepsy; CNS, central nervous system; SE, status epilepticus; mRS, modified Rankin Scale; STESS, status epilepticus severity score; AEDs, antiepileptic drugs; SD, standard deviation.

**Table 2:** Multivariate analysis of predictors for 30-day mortality in SE patients.

Variable	OR	95% CI	*p*
SE etiology			
Acute symptomatic seizures	Reference	–	–
Remote symptomatic unprovoked seizures	0.068	0.006–0.766	0.030
Symptomatic seizures due to progressive CNS disorders	0.236	0.006–9.079	0.438
Seizures of unknown etiology	0.149	0.027–0.807	0.027
mRS baseline	2.582	1.335–4.995	0.005
STESS at SE onset	1.901	1.096–3.298	0.022
Charlson comorbidity index	1.458	0.889–2.391	0.135
Lymphocyte count	0.965	0.637–1.463	0.867
Albumin	0.967	0.856–1.092	0.590
Neutrophil to lymphocyte ratio	1.123	0.940–1.341	0.202
Monocyte to lymphocyte ratio	0.348	0.014–8.879	0.523
PIV	1.000	0.999–1.001	0.768

SE, status epilepticus; OR, odds ratio; CI, confidence interval; CNS, central nervous system; mRS, modified Rankin Scale; STESS, status epilepticus severity score.

### PIV and unfavorable prognosis at 6-month follow-up

Univariate comparisons of clinical information and results of laboratory tests between SE patients with favorable outcomes and those with unfavorable outcomes at 6 months post-SE were presented in Table 3. SE patients with poor prognosis exhibited significantly higher baseline mRS scores, STESS, Charlson comorbidity index, neutrophil count, monocyte count, platelet count, NLR, MLR, PLR, NAR and PIV in comparison to SE patients with good prognosis (all *p* < 0.05). Besides, we observed obviously lower level of albumin in SE patients with poor prognosis compared to SE patients with good prognosis (*p* = 0.026). There was no remarkable difference in sex, age, SE etiology, ratios of SE induced by infection or stroke, the number of AEDs, prior seizures, hemoglobin and lymphocyte count between good prognosis group and poor prognosis group (all *p* > 0.05). Furthermore, the multivariate logistic regression analysis revealed that PIV was strongly related to poor prognosis of SE patients after adjusting for mRS scores, Charlson comorbidity index, hemoglobin, monocyte count, platelet count, PLR, albumin and STESS [odds ratio: 1.002; 95% confidence interval, 1.000–1.004; *p* = 0.026] (Table 4).

**Table 3:** Comparisons of clinical characteristics and laboratory data between SE patients with good-prognosis and poor-prognosis.

Variable	Good-prognosis (*n* = 66)	Poor-prognosis (*n* = 23)	*p*
Male (*n*, %)	41.0 (62.1)	15.0 (65.2)	0.791
Age, years, median (IQR)	50.0 (29.5–60.8)	57.0 (41.5–68.0)	0.167
SE etiology grouped according to the ILAE (*n*, %)			0.909
Acute symptomatic seizures	21.0 (31.8)	7.0 (30.4)	
Remote symptomatic unprovoked seizures	10.0 (15.2)	5.0 (21.7)	
Symptomatic seizures due to progressive CNS disorders	6.0 (9.1)	2.0 (8.7)	
Seizures of unknown etiology	29.0 (43.9)	9.0 (39.2)	
Stroke etiology of SE (*n*, %)	6.0 (9.1)	3.0 (13.0)	0.889
Infectious etiology of SE (*n*, %)	11.0 (16.7)	2.0 (8.7)	0.556
No history of seizures (*n*, %)	42.0 (63.6)	12.0 (52.2)	0.333
mRS baseline, median (IQR)	1.0 (1.0–2.8)	4.0 (1.5–4.0)	0.002
STESS at SE onset, median (IQR)	2.0 (1.3–2.8)	3.0 (2.0–3.5)	0.007
Charlson comorbidity index, median (IQR)	1.0 (0–2.0)	2.0 (1.0–2.5)	0.023
AEDs, median (IQR)	2.0 (2.0–3.0)	3.0 (2.0–3.0)	0.242
Hemoglobin (g/L), mean (SD)	138.0 (18.3)	129.5 (20.9)	0.071
Neutrophil count (x10^3^ /μL), median (IQR)	6.1 (3.9–8.5)	9.2 (5.9–13.4)	0.002
Lymphocyte count (x10^3^ /μL), median (IQR)	1.4 (1.0–2.1)	1.1 (0.8–1.7)	0.100
Monocyte count (x10^3^ /μL), median (IQR)	0.4 (0.3–0.6)	0.7 (0.4–1.1)	0.002
Platelet count (x10^3^ /μL), mean (SD)	212.9 (81.4)	266.6 (86.9)	0.010
Albumin (g/L), mean (SD)	43.1 (4.5)	40.3 (6.6)	0.026
Neutrophil to lymphocyte ratio, median (IQR)	3.4 (2.3–6.8)	7.9 (3.9–14.0)	0.006
Monocyte to lymphocyte ratio, median (IQR)	0.3 (0.2–0.4)	0.5 (0.3–1.1)	0.003
Platelet to lymphocyte ratio, median (IQR)	135.4 (103.4–194.7)	244.3 (167.2–280.6)	0.004
Neutrophil to albumin ratio, median (IQR)	0.1 (0.1–0.2)	0.2 (0.2–0.3)	<0.001
PIV, median (IQR)	328.4 (155.0–644.5)	1280.4 (513.2–2302.8)	<0.001

IQR, interquartile range; ILAE, International League Against Epilepsy; CNS, central nervous system; SE, status epilepticus; mRS, modified Rankin Scale; STESS, status epilepticus severity score; AEDs, antiepileptic drugs; SD, standard deviation.

**Table 4:** Multivariate analysis of predictors for poor outcome in SE patients.

Variable	OR	95% CI	*p*
mRS baseline	1.830	1.093–3.065	0.022
Charlson comorbidity index	1.491	0.923–2.408	0.103
Hemoglobin	1.011	0.973–1.049	0.579
Monocyte count	0.219	0.009–5.523	0.356
Platelet count	1.008	1.000–1.017	0.058
Platelet to lymphocyte ratio	1.000	0.991–1.010	0.925
PIV	1.002	1.000–1.004	0.026
Albumin	0.888	0.757–1.041	0.143
STESS at SE onset	1.205	0.628–2.312	0.575

SE, status epilepticus; OR, odds ratio; CI, confidence interval; mRS, modified Rankin Scale; STESS, status epilepticus severity score.

### The predictive power of PIV for poor outcomes of SE patients

**Figure 1:**
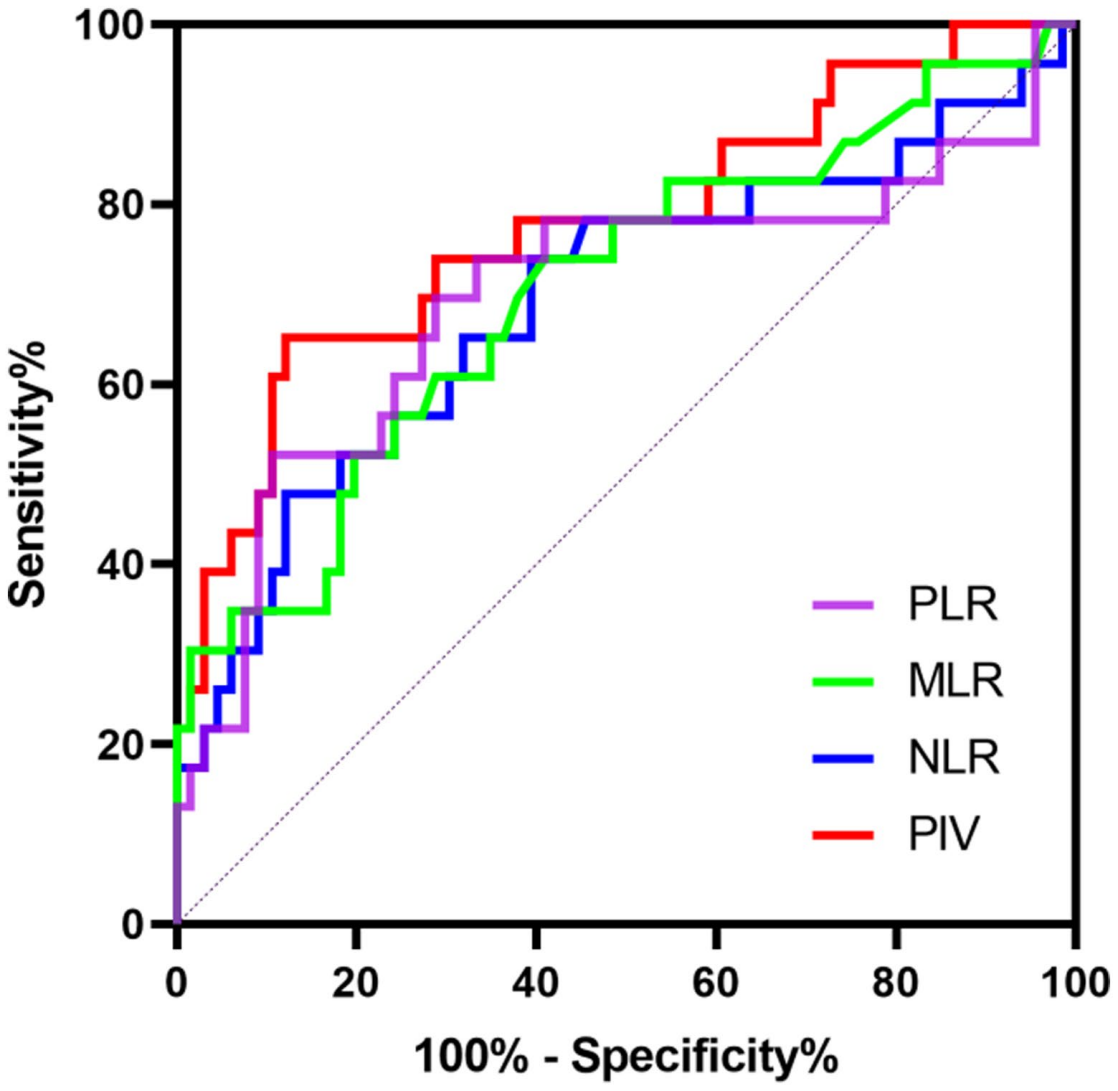
Receiver operating characteristics curve of PIV, NLR, MLR, and PLR to predict poor prognosis in SE patients.

**Figure 2:**
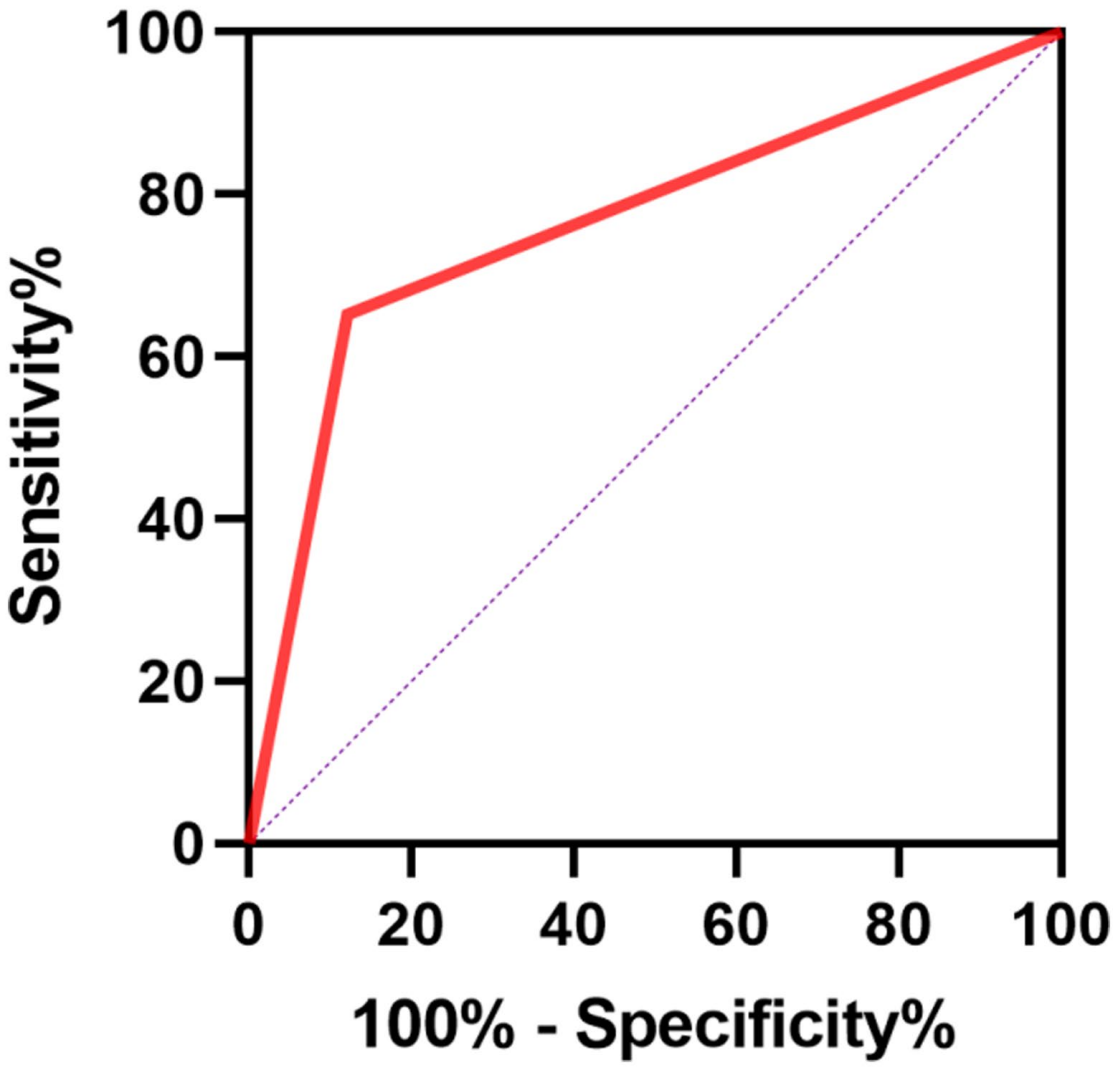
Receiver operating characteristics curve of PIV ≥ 1,090 for predicting poor prognosis in SE patients.

We further conducted the receiver operating characteristic (ROC) curve to explore the predictive ability of PIV for poor outcomes in SE patients. The area under the ROC curve of PIV was 0.777 (95% CI: 0.656–0.897, *p* < 0.001) for poor outcomes, which was larger than those of NLR (0.693, 95% CI: 0.554–0.832, *p* = 0.006), MLR (0.705, 95% CI: 0.573–0.836, *p* = 0.004) and PLR (0.699, 95% CI: 0.554–0.844, *p* = 0.005) (Figure 1), but these differences failed to reach statistical significance (difference between areas PIV vs. NLR: 0.084, 95% CI: −0.010–0.178, *p* = 0.079; difference between areas PIV vs. MLR: 0.072, 95% CI: −0.021–0.165, *p* = 0.129; difference between areas PIV vs. PLR: 0.078, 95% CI: −0.044–0.199, *p* = 0.210). The optimal predictive cutoff value for poor outcomes in SE patients by PIV was 1,090 (sensitivity 65.22%, specificity 87.88%). Furthermore, we evaluated the predictive ability of PIV ≥ 1,090 for poor outcomes in SE patients, which indicated that the area under the ROC curve was 0.766 (95% CI: 0.640–0.891, *p* < 0.001) (Figure 2). Next, 89 patients with SE were classified into two groups according to the PIV cutoff value (PIV < 1,090 and PIV ≥ 1,090). We compared the data of poor 6-month outcome across both groups. Expectedly, a greater proportion of poor 6-month outcome was observed in SE patients with PIV ≥ 1,090 (*p* < 0.001) (Table 5).

**Table 5:** Comparisons of baseline demographic and poor outcome between patients with different levels of PIV.

Variable	PIV		*p*
	< 1,090 (*n* = 66) ≥ 1,090 (*n* = 23)		
Male (*n*, %)	40.0 (60.6)	16.0 (69.6)	0.444
Age, years, median (IQR)	51.5 (31.0–66.5)	50.0 (29.0–62.5)	0.674
SE etiology grouped according to the ILAE (*n*, %)			0.250
Acute symptomatic seizures	17.0 (25.8)	11.0 (47.8)	
Remote symptomatic unprovoked seizures	12.0 (18.2)	3.0 (13.1)	
Symptomatic seizures due to progressive CNS disorders	7.0 (10.6)	1.0 (4.3)	
Seizures of unknown etiology	30.0 (45.4)	8.0 (34.8)	
Stroke etiology of SE (*n*, %)	5.0 (7.6)	4.0 (17.4)	0.346
Infectious etiology of SE (*n*, %)	11.0 (16.7)	2.0 (8.7)	0.556
No history of seizures (*n*, %)	39.0 (59.1)	15.0 (65.2)	0.605
mRS baseline, median (IQR)	1.0 (1.0–4.0)	2.0 (1.0–4.0)	0.109
STESS at SE onset, median (IQR)	2.0 (1.3–3.0)	2.0 (2.0–3.0)	0.714
Charlson comorbidity index, median (IQR)	1.0 (0–2.0)	1.0 (0–2.0)	0.944
AEDs, median (IQR)	3.0 (2.0–3.0)	3.0 (2.0–3.0)	0.762
Poor 6-month outcome (*n*, %)	8.0 (12.1)	15.0 (65.2)	<0.001

IQR, interquartile range; ILAE, International League Against Epilepsy; CNS, central nervous system; SE, status epilepticus; mRS, modified Rankin Scale; STESS, status epilepticus severity score; AEDs, antiepileptic drugs.

### External cohort validated the predictive value of PIV for SE prognosis

We further carried out an external cohort to validate whether PIV ≥ 1,090 was closely correlated with adverse outcomes in SE patients. Univariable and multivariable analyses performed in the external cohort were shown in Tables 6, 7, respectively. Univariable analysis identified baseline mRS scores, STESS, Charlson comorbidity index, the number of AEDs, neutrophil count, monocyte count, lymphocyte count, platelet count, albumin, NLR, MLR, PLR, NAR, PIV, and PIV ≥ 1,090 as prognostic factors for poor outcomes in SE patients (all *p* < 0.10, Table 6). After multicollinearity assessment, Charlson comorbidity index, baseline mRS scores, the number of AEDs, lymphocyte count, platelet count, albumin, NLR, monocyte count, PIV ≥ 1,090, and STESS were included into subsequent multivariable analysis, which demonstrated that PIV ≥ 1,090 was a significant indicator for predicting unfavorable prognosis of SE patients (odds ratio: 42.433; 95% confidence interval, 1.456–1236.343; *p* = 0.029) (Table 7). Hence, the external validation cohort results corroborated the discovery cohort findings, further confirming PIV’s prognostic value in SE patients.

**Table 6:** Comparisons of clinical characteristics and laboratory data between SE patients with good-prognosis and poor-prognosis in the external validation cohort.

Variable	Good-prognosis (*n* = 39)	Poor-prognosis (*n* = 16)	*p*
Male (*n*, %)	21.0 (53.8)	10.0 (62.5)	0.557
Age, years, median (IQR)	67.0 (55.0–75.5)	66.0 (59.8–77.0)	0.505
SE etiology grouped according to the ILAE (*n,* %)			0.906
Acute symptomatic seizures	13.0 (33.3)	6.0 (37.5)	
Remote symptomatic unprovoked seizures	4.0 (10.3)	2.0 (12.5)	
Symptomatic seizures due to progressive CNS disorders	5.0 (12.8)	1.0 (6.3)	
Seizures of unknown etiology	17.0 (43.6)	7.0 (43.7)	
Stroke etiology of SE (*n*, %)	6.0 (15.4)	5.0 (31.3)	0.335
Infectious etiology of SE (*n*, %)	6.0 (15.4)	1.0 (6.3)	0.633
No history of seizures (*n*, %)	23.0 (59.0)	12.0 (75.0)	0.262
mRS baseline, median (IQR)	2.0 (2.0–3.0)	4.0 (2.0–4.0)	0.005
STESS at SE onset, median (IQR)	3.0 (2.0–4.0)	4.0 (2.8–4.0)	0.042
Charlson comorbidity index, median (IQR)	1.0 (0–1.5)	2.0 (1.8–2.8)	0.001
AEDs, median (IQR)	3.0 (2.0–3.0)	3.0 (2.0–4.0)	0.021
Hemoglobin (g/L), median (IQR)	127.0 (119.0–136.5)	128.5 (103.3–134.5)	0.598
Neutrophil count (x10^3^ /μL), mean (SD)	6.9 (2.8)	9.8 (5.2)	0.011
Lymphocyte count (x10^3^ /μL), median (IQR)	1.4 (1.1–1.7)	1.1 (0.5–1.4)	0.047
Monocyte count (x10^3^ /μL), mean (SD)	0.6 (0.2)	0.8 (0.4)	0.032
Platelet count (x10^3^ /μL), mean (SD)	197.7 (72.2)	241.1 (78.7)	0.058
Albumin (g/L), median (IQR)	41.2 (36.3–43.0)	36.9 (33.4–40.7)	0.082
Neutrophil to lymphocyte ratio, median (IQR)	4.7 (2.9–6.6)	7.1 (4.8–16.1)	0.008
Monocyte to lymphocyte ratio, median (IQR)	0.4 (0.3–0.5)	0.6 (0.4–1.2)	0.014
Platelet to lymphocyte ratio, median (IQR)	130.1 (95.2–173.6)	226.6 (147.6–410.6)	0.001
Neutrophil to albumin ratio, mean (SD)	0.2 (0.1)	0.3 (0.2)	0.004
PIV, median (IQR)	447.1 (289.1–767.0)	1236.0 (506.0–3151.4)	0.004
PIV ≥ 1,090 (*n*, %)	4.0 (10.3)	8.0 (50.0)	0.004

IQR, interquartile range; ILAE, International League Against Epilepsy; CNS, central nervous system; SE, status epilepticus; mRS, modified Rankin Scale; STESS, status epilepticus severity score; AEDs, antiepileptic drugs; SD, standard deviation.

**Table 7:** Multivariate analysis of predictors for poor outcome in SE patients in the external validation cohort.

Variable	OR	95% CI	*p*
mRS baseline	1.737	0.547–5.519	0.349
Charlson comorbidity index	1.076	0.515–2.249	0.845
AEDs	1.382	0.181–10.534	0.755
Lymphocyte count	0.264	0.030–2.335	0.231
Platelet count	1.007	0.993–1.022	0.298
Albumin	0.901	0.739–1.097	0.299
Neutrophil to lymphocyte ratio	1.098	0.937–1.287	0.248
Monocyte count	30.812	0.448–2119.960	0.112
PIV ≥ 1,090	42.433	1.456–1236.343	0.029
STESS at SE onset	1.389	0.435–4.440	0.579

SE, status epilepticus; OR, odds ratio; CI, confidence interval; mRS, modified Rankin Scale; AEDs, antiepileptic drugs; STESS, status epilepticus severity score.

## Discussion

Our results indicated that an elevated PIV at admission was closely related to a high risk of poor 6-month prognosis in SE patients. Furthermore, PIV ≥ 1,090 was determined as the critical value to predict unfavorable SE prognosis. Our study suggests that PIV, as a cost-effective and easily available marker, could become a promising predictor for SE prognosis.

There is increasing evidence supporting the involvement of inflammatory and immune mechanisms in the pathogenesis of SE. Following SE onset, substantial inflammatory mediators are released by activated glial cells, further leading to impaired astroglia and neuronal functions, blood–brain barrier (BBB) dysfunction as well as excitation-inhibition imbalance, ultimately resulting in neuronal circuit hyperexcitability and recurrent seizures (Vezzani et al., 2023; Villasana-Salazar and Vezzani, 2023). Additionally, both experimental and clinical studies have demonstrated the activation of innate and adaptive immunity in epileptic brain tissues (Vezzani et al., 2015). Peripheral immune cells entering the brain is an important feature of epilepsy, which may be due to BBB disruption and the recruitment of multiple chemical substances generated by the neurovascular unit (Oby and Janigro, 2006; Tian et al., 2017; Yamanaka et al., 2021). Furthermore, these invading immune cells could promote neuroinflammation and aggravate neuronal injury post-SE, and targeting immune cell brain infiltration represents a potential therapeutic strategy for seizure control (Varvel et al., 2016).

In recent years, the association of immune-inflammatory biomarkers with SE has attracted increased attention. C-reactive protein (CRP) is one of critical circulating markers of inflammation and innate immunity (Metz et al., 2025). A prospective study from Wang et al. (2022) showed a possible correlation between serum CRP level and convulsion SE in children. In addition, Sutter et al. (2015) reported that PCT, an acute-phase protein, was an independent parameter for predicting unfavorable outcomes in SE patients. It is worth noting that evidence on the role of some inflammatory markers in SE prognosis is conflicting. Admission NLR emerged as an independent predictor of in-hospital mortality in patients with generalized convulsive SE (Wang D. et al., 2023; Wang S. et al., 2023). However, another investigation from Olivo et al. (2023) did not find a significant association between NLR at admission and short-term mortality in SE patients. A possible explanation is that SE is an extremely complicated process related to diverse factors including neuronal injury, inflammatory reactions and activation of immune cells, and thus a single parameter may not be adequate to account for the onset and persistence of SE (Shi et al., 2024). PIV, a novel inflammation parameter recently introduced, combines several immune and inflammatory markers and can more comprehensively reflect patients’ inflammatory status, and thus has superior prognostic power than other inflammatory parameters. The research from Fucà et al. (2020) found that elevated PIV showed a robust correlation with reduced progression-free survival (PFS) and overall survival (OS) in metastatic colorectal cancer patients undergoing first-line treatment, and PIV’s prognostic ability outperformed other well-known immune-inflammatory biomarkers including platelet count, monocyte count, NLR and SII. Moreover, Murat et al. (2023) retrospectively enrolled a total of 658 patients with ST-segment elevation myocardial infarction, and observed that PIV had a better predictive value for short and long-term mortality of patients compared to other inflammatory indices including NLR, PLR and SII. In line with previous studies, our data also indicated that PIV was a strong predictor of SE prognosis with better performance than other inflammation markers by using multivariate logistic regression analysis and ROC analysis. Nevertheless, differences in areas under ROC curves between markers did not reach statistical significance, and thus our results warrant additional studies to verify these findings.

Of note, a very recent study from Shi et al. (2024) explored the association of multiple markers including PIV with SE severity assessed by STESS, which revealed no significant correlation between PIV and the severity of SE. As the first prognostic scoring tool for SE, STESS has been widely used to grade the severity and predict the outcome of SE patients (Rossetti et al., 2006). Notably, STESS has been suggested to be only useful for predicting in-hospital death of SE patients but unsuitable for assessing long-term outcome (Aukland et al., 2016). Indeed, our study observed a remarkable association of STESS with 30-day mortality post-SE but no association of STESS with poor 6-month prognosis in SE patients. In contrast, the analysis of the relationship between PIV and SE prognosis obtained opposite results, which indicated that elevated PIV was strongly associated with unfavorable 6-month outcome rather than 30-day death in SE patients. Therefore, the current study suggested that PIV could become a good predictor for relatively long-term outcomes of SE patients. Moreover, the assessment of STESS depends on patients’ seizure history, which is a parameter that cannot be determined if patients have consciousness impairment and their relatives are not present. Comparatively speaking, PIV is a readily available predictor, which could be easily applied in clinical practice.

The current research has several shortcomings. First, this study is based on a retrospective design. Second, we only measured baseline PIV and did not detect the levels of hematological indexes at discharge or during follow-up. Dynamic PIV monitoring may offer more valuable information about the mechanism compared to single-timepoint assessments. Third, the timing of laboratory parameter collection was set to within 24 h post-admission in our study. Due to the difference in the time interval between admission and SE onset, the timing of laboratory examinations relative to SE onset may be different among enrolled patients. The levels of blood routine indicators such as neutrophil count and lymphocyte count may alter dynamically during SE, so future studies can specify the interval between SE onset and laboratory tests, which may contribute to reducing the bias caused by dynamic changes in these indicators at different stages of the disease. Fourth, although we employed the multivariate analysis to adjust potential covariates, residual confounding factors may still influence the results of our study. Fifth, the current study only evaluated the association of PIV with 6-month outcome after SE onset, but more long-term clinical data are needed to further verify our findings.

## Conclusion

Our findings indicated that higher PIV was independently related to unfavorable outcomes of patients with SE. PIV may be a simple, promising and cost-effective predictor for SE prognosis. Further studies are needed to confirm our results and integrate them into clinical practice.

## Data Availability

The datasets presented in this article are not readily available because the data can be obtained through contacting the authors. Requests to access the datasets should be directed to LP, lilei.peng@swmu.edu.cn.
